# In *Sulfolobus solfataricus*, the Poly(ADP-Ribose) Polymerase-Like Thermoprotein Is a Multifunctional Enzyme

**DOI:** 10.3390/microorganisms8101523

**Published:** 2020-10-03

**Authors:** Anna De Maio, Elena Porzio, Sergio Rotondo, Anna Rita Bianchi, Maria Rosaria Faraone-Mennella

**Affiliations:** 1 Department of Biology, Polytechnic School of Basic Sciences, University of Naples “Federico II”, 80126 Naples, Italy; sergio.rotondo@yahoo.it (S.R.); bianchi.annarita@alice.it (A.R.B.); 2 National Institute of Biostructures and Biosystems (INBB), via delle Medaglie d’oro, 00136 Rome, Italy; 3 Institute of Biochemistry and Cell Biology, CNR, via P.Castellino 111, 80131 Naples, Italy; elena.porzio@cnr.it

**Keywords:** *Sulfolobus*, ADP-ribosylation, thermozyme, DING protein, Archaea, Sso7 protein, ATPase, ATP

## Abstract

In *Sulfolobus solfataricus*, Sso, the ADP-ribosylating thermozyme is known to carry both auto- and heteromodification of target proteins via short chains of ADP-ribose. Here, we provide evidence that this thermoprotein is a multifunctional enzyme, also showing ATPase activity. Electrophoretic and kinetic analyses were performed using NAD^+^ and ATP as substrates. The results showed that ATP is acting as a negative effector on the NAD^+^-dependent reaction, and is also responsible for inducing the dimerization of the thermozyme. These findings enabled us to further investigate the kinetic of ADP-ribosylation activity in the presence of ATP, and to also assay its ability to work as a substrate. Moreover, since the heteroacceptor of ADP-ribose is the sulfolobal Sso7 protein, known as an ATPase, some reconstitution experiments were set up to study the reciprocal influence of the ADP-ribosylating thermozyme and the Sso7 protein on their activities, considering also the possibility of direct enzyme/Sso7 protein interactions. This study provides new insights into the ATP-ase activity of the ADP-ribosylating thermozyme, which is able to establish stable complexes with Sso7 protein.

## 1. Introduction

For years, the eukatyotic-like poly(ADP-ribosyl)ation system in the thermophilic *S. solfataricus* was unique [1,2], until the discovery of poly(ADP-ribose) polymerase (PARP) gene orthologues in several prokaryotes [3]. PARP family members catalyse a reversible NAD^+^-dependent post-translational modification of proteins involved in a broad range of cellular processes. The reaction is physiologically regulated by a specific and temporal balance between poly(ADP-ribose) synthesis (by PARPs) and degradation (catalysed by poly(ADP-ribose) glycohydrolases, PARGs) [4,5,6] and references therein.

Up until now, the Sulfolobus thermoprotein (PARPSso) was the best biochemically characterized prokaryotic PARP-like enzyme [1,2]. Like eukaryotic PARPs, the thermozyme elongates the ADP-ribose chain, and binds DNA independently from the sequence, structure and free ends, although it shows a slight preference for a circular structure [1,7,8]. PARPSso is a very stable enzyme, resistant to denaturants including SDS. Moreover, the reaction is reversed by a PARG-like glycohydrolase that has been recently identified and characterised [9].

PARPSso is not structurally related to the known PARPs [10] and localises at the edge of the sulfolobal cell membrane [11].

The thermozyme undergoes automodification by oligo-ADP-ribosylation and has Sso7, the abundant sulfolobal DNA-binding protein, as main heteroacceptor [1,2,7]. The ADP-ribosylated Sso7 protein is unable to condense DNA [12]. This finding allowed us to hypothesize a regulatory role by PARPSso on DNA condensation/decondensation process mediated by Sso7 [12].

It is well known that the group of 7 kDa proteins is highly conserved in all Sulfolobus species, identified with different acronima (Sso, Sac, Sul) [13,14], and they belong to a non-histone group, as compared with histone-like proteins found in other Archaea [13]. The latter form a core which DNA wraps on, whereas Sso7 proteins interact with the minor grooves of DNA, leading to the condensation of nucleic acid [15]. In vitro studies showed that Sso7 promotes annealing of complementary DNA strands [16], induces negative supercoiling [17], and guides the disassembly and renaturation of protein aggregates in an ATP hydrolysis-dependent manner [18]. The different activities are mutually exclusive events [19].

As stated above, Sso7 is the main heteroacceptor of ADP-ribose in *S. solfataricus*, with a consequent inhibition of its ability to condense DNA [8,12].

In previous experiments, the reconstitution of purified PARPSso with homogenous Sso7 in the presence of ^32^P-NAD^+^ in the absence of DNA led to demonstrate that the 7 kDa protein increases the ADP-ribosylating activity of the thermozyme, functioning as an in vitro ADP-ribose acceptor [8]. In other experiments, it was shown that the purified sulfolobal enzyme, incubated with ^32^P-NAD^+^, in the absence of Sso7, had DNA as a positive effector of PARP-like activity, and exhibited a high DNA affinity [7,20].

The aim of the present research was first to study in vitro the effect of PARPSso protein on the ATPase activity of Sso7 too. We present kinetic evidence that ATP, the substrate of ATPase, negatively modulates the ADP-ribosylating activity. Moreover, assaying PARPSso with ATP as a substrate, the thermozyme was able to hydrolyze the nucleotide and had NAD^+^ as an effector. Kinetic parameters of ATPase activity exhibited by PARPSso were also measured. Secondly, we studied whether PARPSso and Sso7 ATPase activities could have a reciprocal influence, and the two proteins would exhibit direct protein-protein interactions.

## 2. Materials and Methods

### 2.1. Materials

[^32^P]NAD^+^, nicotinamide adenine dinucleotide di(triethylammonium)salt (adenylate-^32^P), 1000 Ci/mmol, was purchased by GE Healthcare Europe GmbH (Milano, Italy); [γ-^32^P]ATP (3000 Ci/mmol) was from Perkin Elmer (Milano, Italy). Pre-stained molecular weight markers were purchased from Bio-Rad (Cat No. 1610318, Milano, Italy). NAD^+^, ADP-ribose, ATP, protease inhibitors, and all chemicals were purchased from SIGMA Chemical Company (Milano, Italy). Stock solutions of NAD^+^ and ATP (4 mM) were prepared; concentration was confirmed spectrophotometrically by their molar extinction coefficients. From these solutions, an isotopic dilution was prepared at a given compound concentration and given specific radioactivity (10,000 cpm/ compound nmole) to be used in the assays.

### 2.2. Cell Culture and Homogenate Preparation

*S. solfataricus* strain MT-4 (DSM No. 5833) was grown at 87 °C, in either a small (2.5 L) or large (90 L) fermenter (aeration flux: 30mL/min/L). The standard culture medium, adjusted to pH 3.5 with 0.1 M H_2_SO_4,_ contained (g/L): KH_2_PO_4_, 3.1; (NH_4_)_2_SO_4_, 2.5; MgSO_4_, 7H_2_O, 0.2; CaCl_2_, 2H_2_O, 0.25, yeast extract, 2. Sometimes, in the standard medium, yeast extract was replaced by glucose (3 g/L) as the carbon source. This condition was used to avoid any possible protein/nucleic acid contamination from yeast. Cells were harvested in the stationary phase of growth, at a concentration of 0.5 g of freeze-dried cells/L by continuous flow centrifugation in an Alfa Laval model LAB 102 B20 centrifuge. After two washes in an iso-osmotic saline solution (pH 6.0), they were collected by centrifugation at 9000× *g* for 30 min. Cells were stored at −20 °C for several months without loss of enzymatic activities.

The crude homogenate was prepared from the collected cells as described in [1]. A cocktail of protease inhibitors was added to all protein solutions (2 μg/mL; containing 20 μg/mL pancreas-extract, 0.5 μg/mL thermolysin, 2 μg/mL chymotrypsin, 2 μg/mL trypsin, 330 μg/mL papain).

### 2.3. Purification and Electrophoresis of PARPSso and Sso7 Proteins

PARPSso was purified by means of a two-chromatographic step protocol, including two affinity chromatographies [20]. The purified enzyme used in the described experiments was from three different preparations, one freshly prepared from cells grown in a 2.5 L fermentor, two others stored at −20 °C for two-three months. The freshly prepared enzyme was used in parallel with one of the two frozen purified PARPSso, that had comparable activities. The purification of the Sso7 protein was achieved by using a 5% perchloric acid extract of sulfolobal homogenate, followed by the procedure previously described [8]. Protein concentration was determined by the Bradford procedure according to [1].

The homogeneity of the two proteins was checked by SDS-PAGE (12%) and silver staining of the gel [12].

### 2.4. SDS-PAGE and Western Blot of Purified PARPSso

The electrophoretic analysis of the purified PARPSso fractions was carried out on a polyacrylamide gel (12%) by means of a Tris-glycine-0.1%SDS system, pH 8.3 at 18 mA [1]. The electrophoretic analysis was carried out by using duplicate aliquots of purified PARPSso in different experimental conditions. After electrophoresis, one of each duplicate was stained with 0.1% Coomassie Brilliant Blue R and then destained in a 10% acetic acid (*v*/*v*)/10% methanol solution (*v*/*v*). The proteins of the unstained gel (second duplicate) were electrotransferred onto poly(vinylidene difuloride) (PVDF) membrane filter (0.45 μm; Cat No. IPVH00010, Merck Millipore, Milano, Italy) by a Bio-Rad Transblot system at constant 200 mA in 0.025 M Tris–0.192 M glycine buffer, pH 8.6, containing SDS 0.025% at 4 °C for 2 h. The filter underwent repeated washes with 50 mM Tris-HCI buffer, pH 8.0, and 150 mM NaCl (TBS), containing 0.5% Tween, and then incubated at room temperature for 3 h, in TBS-0.5% Tween, containing 3% gelatine to saturate the non specific bond sites. Thereafter, the filter was incubated at room temperature, overnight, in the presence of anti-PARP catalytic site polyclonal antibodies (rabbit anti-human PARP, H-250, 1:1000, *v*/*v*; Cat No. sc7150, Santa Cruz Biotechnology, Heidelberg, Germany) in 0.05% TBS-Tween and 0.3% gelatine. After several washes in TBS-0.05% Tween, at room temperature for 1 h, PVDF filter was incubated with peroxidase (HRP)-conjugated goat anti-rabbit (Cat No. 31460, Life Technologies, Monza, Italy) directed against the primary antibodies.

Finally, the filter underwent a series of washes, before detecting peroxidase activity by chemiluminescence, using a Pierce kit (Super Signal West Dura, Pierce, Life Technologies, Monza, Italy).The acquisition and analysis of the images were carried out by Chemidoc (Bio-Rad) and the Quantity One program.

### 2.5. PARPSso Assay

Standard activity assay of purified PARPSso (10 μg) was carried out in the presence of 0.64 mM [^32^P]NAD^+^ (10,000 cpm/nmol) and according to [1,12]. Briefly, the reaction mixture (final volume 62.5 μL), contained [^32^P]NAD^+^, 0.1 M Tris-HCl buffer (pH 7.5), and 4 mM NaF, as glicohydrolase inhibitor. Where [^32^P]NAD^+^ final concentration changed, the [^32^P] isotopic dilution was 10,000 cpm/nmol. Incubation in sealed vials was at 80 °C for 10 min, unless otherwise stated.

After incubation, the reaction was blocked on ice, by adding 25% TCA (*v*/*v*), to precipitate the proteins. The following washes of precipitate on Millipore filters (0.45 μm; Cat No. HAWP00010, Merck Millipore, Milano, Italy) with cold 7% TCA (20–30 volumes), removed free NAD^+^; the protein-associated radioactivity on filter was measured in a liquid phase scintillator (BECKMAN LS 1701), in the scintillation liquid (LIPOFLUOR, LUMA).

The activity was also assayed in the presence of different concentrations of ATP (5 µM–100 µM) and Sso7 (10 µg). The activity values are means of four determinations with the purified enzyme from two preparations.

The enzymatic activity was expressed in mUnits; 1 mU is the enzyme amount required to convert 1 nmole NAD^+^ into ADPR in one minute, at pH 8.0 and at 80 °C.

### 2.6. ATPase activity of PARPSso and SSo7

ATPase activity was assayed by incubating purified PARPSso (10 μg) in a mixture of 5 mM MgCl_2_, 50 mM sodium phosphate, pH 7.5, in the presence of [γ-^32^P]ATP (10,000 cpm/nmol), for 5 min at 70 °C, in a final volume of 150 μL. The final ATP concentrations changed as in the figures. The [^32^P] isotopic dilution was 10,000 cpm/nmol for all. The activity was also assayed in the presence of two concentrations of NAD^+^ (10 μM and 100 μM) or Sso7 (10 μg). As a control, the ATPase activity of SSo7 protein, already described [14], was assayed. The specific composition of reaction mixtures is reported in the legends of figures. After incubation, duplicate aliquots (25 μL), were drawn from the assay mixture, added to 0.5 mL of a suspension containing 50 mM HCl, 5 mM H_3_PO_4_ and 7% activated charcoal, and centrifuged at 4000× *g* for 20 min, as reported in [18]. The radioactivity (free [^32^P]) of the supernatant was determined from a 100-µL aliquot. For rate calculations, corrections were made for the amount of spontaneous ATP hydrolysis, in the absence of either one or the other protein (blank). The values shown in the figures are means of four determinations with the purified enzyme from two preparations.

Enzymatic unit is expressed as nanomoles of free phosphate produced in one minute.

### 2.7. PARPSso/Sso 7 Interactions

Protein mixtures were prepared at different PARP*Sso*/Sso7 ratios in 10 mM Na-phosphate buffer/10 mM EDTA, pH 7.5, in sealed vials (final volume 10 μL) and incubated at 80 °C for 20 min. The experiment was carried on twice at 80 °C and once at 45 °C. After incubation the samples were transferred on ice, diluted with sample buffer (2×; *v*/*v*) and analysed by SDS-PAGE (5–12% polyacrylamide gradient) [21]. Gels were silver stained [21].

### 2.8. Data Base Analysis

Amino acid sequence and structural motifs were analysed by ExPasy Bioinformatic resource [22]. Secondary structure prediction was according to Ashok Kumar [23].

### 2.9. Statistics

Standard deviations were calculated by Excel program. Graphics were drawn by Graph Pad program. Mann–Whitney U test allowed one to calculate the significance differences (*P*) [24].

## 3. Results

### 3.1. Effect of ATP on S. solfataricus ADP-Ribosylating Thermozyme

Basic kinetic with NAD^+^ as a substrate was described by a sigmoid curve, typical of a cooperative process (Figure S1). The linearizing Hill plot confirmed the cooperativity and allowed one to calculate the Hill coefficient, (*n*_H_ = 1.8), Figure S1 (inlet).

**Figure 1:**
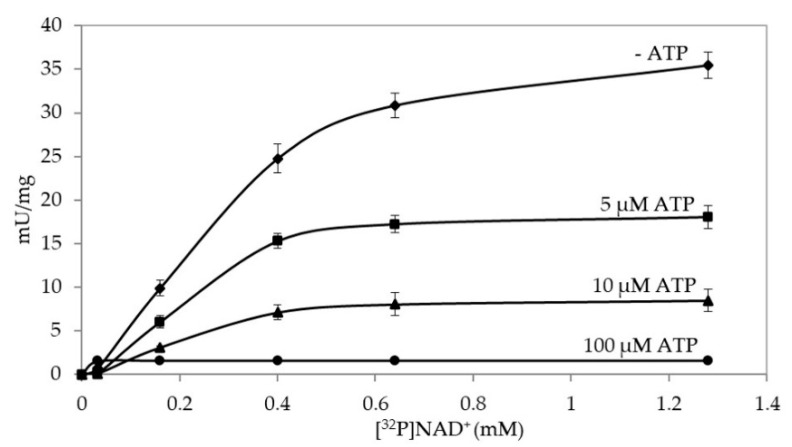
ATP effect on PARPSso activity. Polymerase activity was assayed with [^32^P]NAD^+^ as described in § 2.5, in presence of different ATP concentrations (µM: 0, ♦; 5, ■; 10, ▲; 100, ●). The basic kinetic curve (♦) is the one described in Figure S1.

In order to assess the effect of ATP on PARPSso, the same kinetic was carried out by measuring PARPSso activity, either in the absence or in the presence of ATP (5 μM, 10 μM and 100 μM) at increasing NAD^+^ concentrations (Figure 1).

All kinetics were described by sigmoid curves, indicating ATP as an activity modulator. In detail, ATP behaved as a negative effector, inhibiting PARPSso activity, already at the lowest concentration (5 μM), where specific activity was highly influenced; in fact, at K_0.5_ (0.4 mM NAD^+^), it was reduced by almost 40%. The inhibitory effect was dose-dependent with a maximum at 100 µM nucleotide. At this concentration K_0.5_ splitted to 0.5 mM NAD^+^ and the specific activity was close to zero. Linearizing the plots, the trend was close to that of a mixed inhibition, were both K_M_ and V_max_ changed (Figure S2). This behaviour might be explained by considering that the ADP-ribosylating reaction is a three-step reaction with two substrates, NAD and the protein acceptor of ADP-ribose. Under the kinetic condition, used ATP interferes only with NAD binding site. In the binding site both NAD^+^ and ATP share in part the binding domain, as it is probably different in the region binding the pyridinic nucleotide. The sigmoidal curves determined in the basic kinetic (only NAD^+^ present), were typical of oligomeric and allosteric proteins, and had been described also for eukaryotic PARP1. It was suggested that the dimerization of this enzyme is a prerequisite to the automodification reaction ([25] and references therein). Thus, it was conceivable that the allosteric kinetic of *Sulfolobus* PARPSso could depend on the dimerization of the thermozyme. In fact, a previous electrophoretic experiment showed that a dimeric enzyme occurred upon incubation of PARPSso with the pyridinic substrate [21]. This result was detected also in some Sulfolobus crude preparations where monomeric PARPSso (46.5 kDa), and at least its dimer occurred naturally [21]. Trypsin digestion of the 90 kDa protein and amino acid sequence analysis of peptides by mass spectrometry further demonstrated the identity of monomeric and dimeric PARPSso [26].

### 3.2. PARPSso Dimerization in Presence of ATP

**Figure 2:**
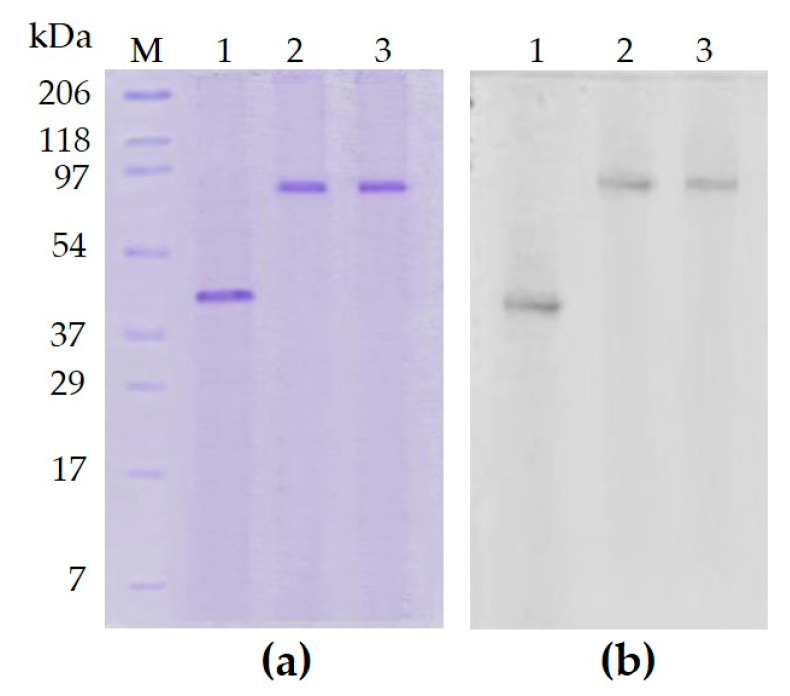
PARPSso dimerization in the presence of NAD^+^ and ATP. (**a**) SDS-PAGE (12%) and staining in 0.1% Comassie brilliant blue R; (**b**) immunoblotting with anti-PARP antibody (Santa Cruz, rabbit anti-human PARP, H-250, 1:1000, *v*/*v*). Incubation conditions in presence of NAD^+^ and ATP are reported in § 2.4. Lanes: 1, purified PARPSso (20 µg); 2, purified PARPSso (20 μg) incubated in standard assay conditions for polymerase activity); 3, as in 2, in presence of 4 mM ATP. M, prestained molecular weight markers (Bio-Rad): myosin (206 kDa), beta-galactosidase (118 kDa), bovine serum albumin (97 kDa), ovalbumine (54 kDa), carbonic anhydrase (37 kDa), trypsin inhibitor (29 kDa), lysozyme (17 kDa), aprotinin (7 kDa). The experiment was carried out also on 0.4 mM compounds with similar results.

On the basis of the results described in the previous section, we set up a similar electrophoretic experiment to study whether ATP could also induce PARPSso dimerization. Figure 2 shows the results of SDS-PAGE and anti-PARP1 immunoblotting in the absence and presence of either NAD^+^ or ATP in standard assay conditions.

In the presence of either compound, the purified monomer splits to dimer, with the disappearance of the band at 46.5 kDa, and the appearance of a signal at 92 kDa. On one side, these results confirmed that enzyme dimerization induced by NAD^+^ (Figure 2, lane 2) could account for the allosteric kinetic of PARPSso (Figure 1). On the other hand, they evidenced that ATP induced the same PARPSso dimerization as NAD^+^. Moreover, the fact that the results were similar by incubating PARPSso with ten times less (0.4 mM) nucleotides evidences that dimerization event occurs even at very low concentration of both compounds.

It must be underlined that PARPSso is not structurally related to the canonical PARP members; it belongs to a serendipitously discovered protein family, with a common N-terminus (DINGGG), a highly conserved phosphate-binding site, and a Walker A motif, binding nucleoside triphosphates (NTP) [26,27,28,29].

### 3.3. ATPase Activity of S. solfataricus PARPSso

The next question to solve was to study whether ATP might be itself a substrate of PARPSso.

The basic kinetic, carried on with ATP as a substrate, was described by a sigmodal curve (Figure S3). Linearization by Hill equation gave a plot showing cooperativity with *n*_H_ = 3.1 (inlet of Figure S3). This result gave evidence that the thermozyme had also ATPase activity.

**Figure 3:**
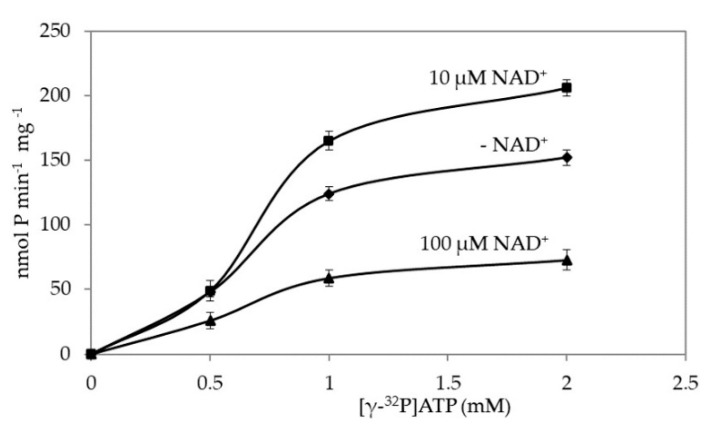
ATPase kinetic of PARPSso. Assays are described in § 2.6. NAD^+^ added at different concentrations (µM: 0, ♦; 10, ■; 100 µM, ▲). The basic kinetic curve (♦) is the one described in Figure S2.

In the presence of NAD^+^, the kinetic followed sigmoidal trends too (Figure 3).

Furthermore, in this case, the allosteric kinetic could be explained by PARPSso dimerization induced by ATP (Figure 2, lane 3). 

The basic kinetic (♦, Figure 3), reached 50% saturation at fairly 0.6 mM ATP (K_0.5_). In the presence of 10 µM NAD^+^, this value increased to 0.75 mM ATP, with a 10–15% increase of specific activity. Moreover, activity inhibition was measured above 0.5 mM ATP. Up to 0.5 mM ATP, the two curves overlapped.

At 100 µM NAD^+^, nearly 20% activity was still measured. At this effector concentration inhibition of ATPase was more evident.

Therefore, NAD^+^, at a low concentration shows an enzyme activation, but at the highest concentration works as an allosteric inhibitor of ATPase activity, even if its effect is lower than the same concentration of ATP on the ADP-ribosylation activity (Figure 1). It was worth nothing that linearizing the plots for ATPase activity in absence and presence of NAD, inhibition was a typical competitive one, varying only K_M_ (Figure S4). This is not surprising as ATPase reaction is simpler than ADP-ribosylation one: a single substrate in competition with the effector NAD^+^. Thus, the binding of ATP to the AMP moiety of the active site is modulated by the concentration of NAD^+^: increasing this compound, its binding is favoured and ADP-ribosyation is the reaction going on. At very low NAD^+^ concentration and large ATP amount, ATPase activity preveals.

A comparison of ADP-ribosylation and ATPase kinetics of PARPSso indicated a *k_cat_* with ATP (140 s^−1^) as a substrate, seven times higher than that with NAD^+^ (*k_cat_* = 20 s^−1^). Catalytic specificities (k_cat_ /K_0.5_) were 230 (ATP) versus 51 (NAD^+^).

### 3.4. Polymerase and ATPase Activity of Thermozyme in Presence of Sso7 Protein

**Figure 4:**
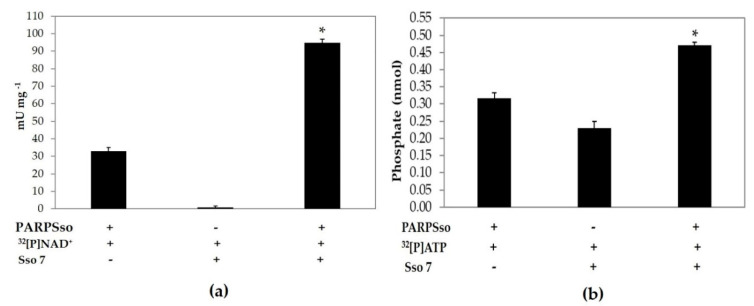
Sso7 effect on polymerase and ATPase activity of thermozyme. (**a**): Polymerase activity was assayed in standard condition, as reported in § 2.5 by incubating: (i) purified PARPSso (10 µg); (ii) purified Sso7 (10 μg); (iii) purified PARPSso (10 µg) in presence of purified Sso7 (10 μg). (**b**): ATPase activity was assayed as described in § 2.6 by incubating: (i) thermozyme (10 µg); (ii) Sso7 (10 μg of proteins); (iii) purified thermozyme (10 µg) in presence of purified Sso7 (10 μg). The values were the means of four different experiments in duplicate. The significance differences (*: *p* < 0.05) were tested with the Mann–Whitney U test [24].

Since it is well known that Sso7 is not only the preferred hetero acceptor of poly-ADPR, but also possesses an ATPase activity [18], we studied whether and how SSo7 could influence the ADP-ribosylation and ATPase activities of PARPSso (Figure 4).

ADP-ribosylation activity of PARPSso in presence of Sso7 produced a three times increase of basal activity, whereas Sso7 incubated alone with ^32^[P]NAD^+^ was not able to use the pyridinic compound as a substrate (Figure 4a). Thus, the activity increase observed upon incubation of PARPSso with the 7 kDa protein, was exclusively due to ADP-ribosylation of Sso7.

On the other hand, both proteins exhibited a significant ATPase activity, and it was more pronounced for PARPSso (Figure 4b).

The simultaneous presence of PARPSso and Sso7 in ATPase standard assay mixture gave an activity increase which was the sum of those exhibited separately by the two proteins (Figure 4b). This result indicated that there was no reciprocal interference in ATPase activities of the two enzymes, independent each other. On the contrary, the three times increase of ADP-ribosylation activity was induced by the presence of Sso7, the target protein of PARPSso heteromodification.

### 3.5. PARPSso/Sso7 Protein Interactions

**Figure 5:**
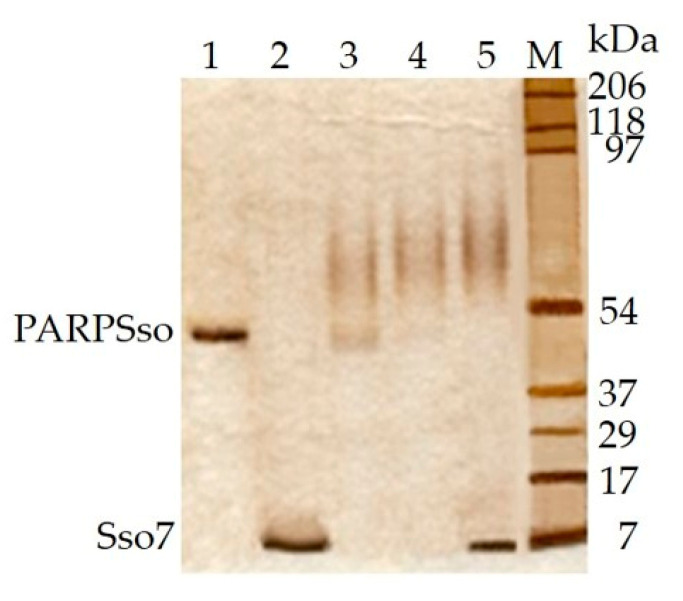
PARPSso and Sso7 interactions. Complexes of the two proteins were evidenced by SDS-PAGE (5–12% gradient) and silver staining of the gel [21]. Lanes: 1, purified PARPSso (3 µg); 2, purified Sso7 protein (1.2 µg, 171 pmoles); 3–5, purified PARPSso (3 µg, 65 pmoles) in the presence of increasing concentrations of Sso7 (1:0.5, 1:1, 1:3, mol/mol). M, pre-stained molecular weight markers (Bio-Rad), as in Figure 2.

Another matter to be further explored was to ascertain whether Sso7 as substrate of ADP-ribosylation might establish protein-protein interactions with PARPSso. Figure 5 shows the electrophoretic patterns of the two purified proteins analysed separately (lanes 1, 2) and incubated in a mixture at different molar ratios (lanes 3–5) under the conditions described in Materials and Methods.

The molecular weight shift of PARPSso monomer (46.5 kDa) in the presence of Sso7 suggested that the two proteins interact with each other by forming complexes at molecular weight as much higher, as much Sso7 concentration increased. In detail, in Figure 5 the silver stained basal bands at 46.5 and 7 kDa (lanes 1, 2 respectively), tended to disappear in lanes 3 and 4, and were replaced by other signals below 90 kDa, corresponding to intermediate protein-protein complexes. In lane 5, the whole PARPSso was used to form complexes, whereas a net Sso7 band appeared again at 7 kDa, indicating that saturation was achieved in the complexes. At this ratio (PARPSso/Sso7 ratio 1:3, mol/mol), the complexes were evident up to the protein marker at 90 kDa. By considering that in lane 4 the complex almost reaches saturation (Sso7 disappears), the ratioSso7 (1.2 µg, 171 pmol)/PARPSso (3 µg, 65 pmol) is close to 3.

## 4. Discussion

The results presented here clearly show that PARPSso exhibits two enzymatic activities. It is able to modify itself and a heteroacceptor, the Sso7 protein, by ADP-ribosylation, or to act as an ATPase, being the ATPase activities of PARPSso and Sso7 protein, independent each other.

**Figure 6:**
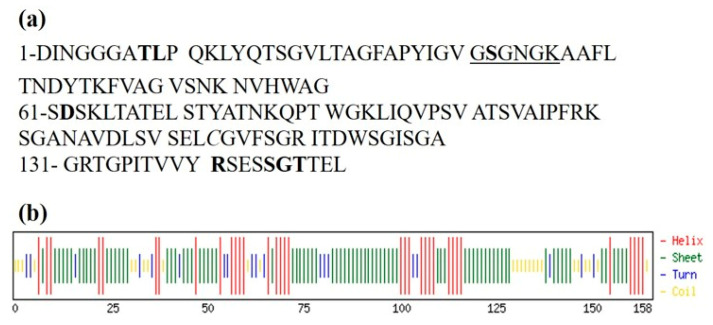
Partial amino acid sequence of PARPSso. (**a**) Amino acids defining the phosphate binding (P)-site are in bold. PARPSso accession number *B3EWG9* (UniProt). The Walker A motif is underlined. (**b**) Prediction of secondary structure of PARPSso [23]. The predicted secondary structure of this region indicates the cluster of beta-sheets flanked by alfa-helices described for other DING proteins.

The ATPase activity of Sso7 had been widely described [17], and confirmed in our experiments, demonstrating also that both Sso7 and PARPSso are ATPase with a mutually exclusive activity (Figure 4). It is worth noting that the ATPase kinetic of PARPSso is modulated by NAD^+^ as a competitive inhibitor, which shares in part with ATP the moiety to bind the active site (Figure S4). The ATPase inhibition trend accounts for a simple reaction catalysed by PARPSso, which probably occurs in the cell at zero or very low NAD^+^ levels playing a different role than the ADP-ribosylating reaction. We highlight that, in the cell, PARPSso is localized along the membrane and kept in touch with both it and the nucleoid [11]. This observation is important because PARPSso was first discovered as an ADP-ribosylating enzyme, catalysing a three-step reaction more complex than the hydrolysis of ATP. Structurally, PARPSso belongs to the family of DING proteins, and similarly to other members of the DING family, it is a monomer, and has a single highly unvariant phosphate-binding site (P-loop, Figure 6). 

The eight conserved residues of P-loop are T8, L9, S32, D62, R141, S145, G146, T147 (Figure 6a) [10,26]. Previously, we demonstrated that, thanks to this site, PARPSso, as monomer, exhibits also phosphatase activity, as other DING proteins from different organisms [26]. What is relevant to discuss here is how PARPSso, a monomer with a P-loop, can catalyse reactions like hydrolysis of ATP, and moreover, a three-step reaction like ADP-ribosylation. Here, we demonstrate that the presence of either one or the other substrate (ATP or NAD^+^) induces the dimerization of PARPSso, suggesting common structural binding motifs. It is widely reported that many ABC ATPases, first discovered as part of membrane transporter systems [27,28,29,30,31], have P-site and function as dimers. This P-site binds phosphate through hydrogen bonds and encloses the Walker A motif (consensus sequence GXXXXGKT/S) [27]. The lysine residue in the consensus GXXXXGKT/S is crucial for the direct interaction with the phosphates of ATP. Similar to ABC ATPases, PARPSso has P-site and Walker A motifs enclosed in an α/β motif at N-terminus, also exhibits highly cooperative ATP-hydrolysis, and functions as a dimer [21,25]. This structural similarity allows one to explain its ATPase activity: the ATP binding site of ABC-ATPases is present in PARPSso too.

How to explain the more complex ADPribosylating activity of PARPSso? This activity of PARPSso requires NAD^+^ as a substrate. NAD^+^ induces the dimerization of PARPSso. The sigmoidal curves determined in the basic kinetic (only NAD^+^ present, Figure 1) were typical of oligomeric and allosteric proteins, and had been described also for eukaryotic PARP1. It was suggested that the dimerization of this enzyme is a prerequisite to the intermolecular automodification reaction ([25] and references therein). Thus, it is conceivable that the allosteric kinetic of *Sulfolobus* PARPSso could depend on the dimerization of the thermozyme. Here, we demonstrate that the thermozyme dimerizes in the presence of NAD^+^, and it suggests a head-to-tail arrangement of two protein molecules forming the active site. Thus, dimerization plays an important role in both catalytic activities of PARPSso. Looking at structural motifs contributing to the active site, as said above, PARPSso has P-site and Walker A motif enclosed in an α/β motif at N-terminus, and functions as a dimer, probably with a head-to-tail arrangement of two thermozyme molecules [21,26]. These motifs, which account for ATPase activity, have been described also in DNA repair enzymes Rad50 and MutS and SMC proteins [32], where two molecules work in pair, by a similar dimer arrangement. The head-to-tail arrangement of such a dimer forms, at the interface, two functional active sites [32].

On the other hand, the proteins that bind to nucleotide cofactors such as NAD(P) usually adopt the Rossmann fold motif. [33]. The GXXXG/A amino acid stretch of Rossmann fold β/αmotif is within the dinucleotide binding domain and stabilizes helix–helix interactions in both membrane and soluble proteins. Specifically, nicotinamide adenine dinucleotide (NAD^+^)-utilizing enzymes share a Gly-rich loop that interacts with the cofactors phosphate moieties. The N-terminus of PARPSso encloses these amino acid and secondary structure motifs (Figure 6) [10,26].

In summary, both P-loop nucleoside-triphosphatase (NTPase) fold and the Rossmann fold-proteins belong to the class of β/α proteins and share a Gly-rich loop. They are among the most widely occurring protein folds presumed to exist in the last universal common ancestor and considered as the earliest precursors of modern proteins [33]. It is worth noting that, in proteins with a similar architecture, both Rossmann fold and ATP-binding sites have been identified [33]. Both Rossmann- β/α fold and P-loop were also identified in RuvBL1, an evolutionarily highly conserved eukaryotic protein belonging to the AAA-family of ATPases (ATPase associated with diverse cellular activities), involved also in chromatin remodelling, DNA repair and apoptosis [34].

On this basis, the co-presence in PARPSso of structural motifs able to bind mono- and dinucleotides within β/α modules might allow one to explain the double activity of the thermozyme.

PARPSso can be numbered among multifunctional proteins, often reported in literature [35,36,37]. The ability to catalyse different activities is an important tool, especially in prokaryotes, to expand their limited amount of genomic information [35,36,37]. At the molecular level, the catalytic promiscuity of a single domain, that is, its ability to catalyse both a primary substrate-specific function and a different, secondary reaction, can account for multifunctionality.

Studies are in progress to assess whether hydrolysis of ATP and protein ADP-ribosylation take part to the same regulatory mechanism or are involved in different processes.

## Supplementary Materials

The following are available online at https://www.mdpi.com/2076-2607/8/10/1523/s1, Figure S1: PARPSso Activity; Figure S2: Linearized plots of ADP-ribosylating activity in the presence of ATP; Figure S3: ATPase activity of PARPSso; Figure S4: Linearized plots of ATPase activity in the presence of NAD^+^.

## References

[1] Faraone-Mennella M.R., Gambacorta A., Nicolaus B., Farina B. (1998). Purification and biochemical characterization of a poly(ADP-ribose) polymerase-like enzyme from the thermophilic archaeon *Sulfolobus solfataricus*. Biochem. J..

[2] Faraone Mennella M.R., Castellano S., De Luca P., Discenza A., Gambacorta A., Nicolaus B., Farina B. (2000). Comparison of the ADP-ribosylating thermozyme from *Sulfolobus solfataricus* and the mesophilic poly(ADP-ribose) polymerases. FEMS Microbiol. Lett..

[3] Otto H., Reche P.A., Bazan F., Dittmar K., Haag F., Koch-Nolte F. (2005). In silico characterization of the family of PARP-like poly(ADP-ribosyl)transferases (pARTs). BMC Genom..

[4] Li N., Chen J. (2014). ADP-ribosylation: Activation, recognition, and removal. Mol. Cells.

[5] Hassa P.O., Hottiger M.O. (2008). The diverse biological roles of mammalian PARPs, a small but powerful family of poly-ADP-ribose polymerases. Front. Biosci..

[6] Luo X., Kraus W.L. (2012). On PAR with PARP: Cellular stress signaling through poly(ADP-ribose) and PARP-1. Genes Dev..

[7] Faraone-Mennella M.R., De Luca P., Giordano A., Gambacorta A., Nicolaus B., Farina B. (2002). High stability binding of poly(ADPribose) polymerase-like thermozyme from *S. solfataricus* with circular DNA. J. Cell. Biochem..

[8] Faraone-Mennella M.R., Farina B. (1995). In the thermophilic archaeon *Sulfolobus solfataricus* a DNA-binding protein is in vitro (ADP-ribosyl)ated. Biochem. Biophys. Res. Commun..

[9] De Maio A., Porzio E., D’Angelo R., Rotondo S., Bianchi A.R., Confalone E., Raucci R., Natale E., Faraone-Mennella M.R. (2015). A Glycosyltransferase from *Sulfolobus solfataricus* MT-4 Exhibits Poly(ADP-ribose) Glycohydrolase Activity. Curr. Proteom..

[10] Di Maro A., De Maio A., Castellano S., Parente A., Farina B., Faraone-Mennella M.R. (2009). The ADP-ribosylating thermozyme from *Sulfolobus solfataricus* is a DING protein. Biol. Chem..

[11] Porzio E., Bianchi A.R., Baccigalupi L., Isticato R., Faraone Mennella M.R. (2016). The DINGGG thermoprotein is membrane bound in the Crenarchaeon *Sulfolobus solfataricus*. Chem. Biol. Technol. Agric..

[12] Castellano S., Farina B., Faraone-Mennella M.R. (2009). The ADP-ribosylation of *S. solfataricus* Sso7 modulates protein/DNA interactions in vitro. FEBS Lett..

[13] Pereira S.L., Grayling R.A., Lurz R., Reeve J.N. (1997). Archaeal nucleosomes. Proc. Natl. Acad. Sci. USA.

[14] Mc Afee J.G., Edmondson S.P., Datta P.K., Shriver J.W., Gupta R. (1995). Gene cloning, expression and characterization of the sac 7 proteins from the Hyperthermophile *S. acidocaldarius*. Biochemistry.

[15] Agback P., Baumann H., Knapp S., Ladenstein R., Härd T. (1998). Architecture of non-specific protein-DNA interactions in the Sso7d-DNA complex. Nat. Struct. Biol..

[16] Guagliardi A., Napoli A., Rossi M., Ciaramella M. (1997). Annealing of complementary DNA strands above the melting point of the duplex promoted by an archaeal protein. J. Mol. Biol..

[17] Napoli A., Zivanovic Y., Bocs C., Buhler C., Rossi M., Forterre P., Ciaramella M. (2002). DNA bending, compaction and negative supercoiling by the architectural protein Sso7d of *Sulfolobus solfataricus*. Nucleic Acids Res..

[18] Guagliardi A., Cerchia L., Moracci M., Rossi M. (2000). The chromosomal protein Sso7d of the crenarchaeon *Sulfolobus solfataricus* rescues aggregated proteins in an ATP hydrolysis-dependent manner. J. Biol. Chem..

[19] Guagliardi A., Cerchia L., Rossi M. (2002). The Sso7d protein of *Sulfolobus solfataricus*: In vitro relationship among different activities. Archaea.

[20] Faraone-Mennella M.R., Piccialli G., De Luca P., Castellano S., Giordano A., Rigano D., De Napoli L., Farina B. (2002). Interaction of the ADP-ribosylating enzyme from the hyperthermophilic archaeon *S. solfataricus* with DNA and ss-deoxyribonucleorides. J. Cell. Biochem..

[21] De Maio A., Porzio E., Romano I., Nicolaus B., Faraone Mennella M.R. (2011). Purification of the poly-ADP-ribose polymerase-like thermozyme from the archaeon *Sulfolobus solfataricus*. Methods Mol. Biol..

[22] ExPASy Bioinformatics Resource Portal. http://www.expasy.org.

[23] Ashok Kumar T. (2013). CFSSP: Chou and Fasman secondary structure prediction server. Wide Spectr..

[24] Whitney J. (1997). Testing for differences with the non parametricMann-Whitney U test. J. Wound Ostomy Cont. Nurs..

[25] Kun E., Kirsten E., Mendeleyev J., Ordahl C.P. (2004). Regulation of the enzymatic catalysis of poly(ADP-ribose) polymerase by dsDNA, polyamines, Mg^2+^, Ca^2+^, histones H1 and H3, and ATP. Biochemistry.

[26] Porzio E., De Maio A., Ricciardi T., Mistretta C., Manco G., Faraone-Mennella M.R. (2018). Comparison of the DING protein from the archaeon *Sulfolobus solfataricus* with human phosphate-binding protein and *Pseudomonas fluorescence* DING counterparts. Extremophiles.

[27] Berna A., Scott K., Chabriere E., Bernier F. (2009). The DING family of proteins: Ubiquitous in eukaryotes, but where are the genes?. Bioessays.

[28] Bernier F. (2013). DING proteins: Numerous functions, elusive genes, a potential for health. Cell. Mol. Life Sci..

[29] Kirschner K., Bisswanger H. (1976). Multifunctional proteins. Annu. Rev. Biochem..

[30] Schmitt L., Tampé R. (2002). Structure and mechanism of ABC transporters. Curr. Opin. Struct. Biol..

[31] Hopfner K.P., Tainer J.A. (2003). Rad50/SMC proteins and ABC transporters: Unifying concepts from high-resolution structures. Curr. Opin. Struct. Biol..

[32] Groothuizen F.S., Sixma T.K. (2016). The conserved molecular machinery in DNA mismatch repair enzyme structures. DNA Repair.

[33] Kleiger G., Eisenberg D. (2002). GXXXG and GXXXA motifs stabilize FAD and NAD(P)-binding Rossmann folds through C(alpha)-H... O hydrogen bonds and van der waals interactions. J. Mol. Biol..

[34] Matias P.M., Gorynia S., Donner P., Carrondo M.A. (2006). Crystal structure of the human AAA+ protein RuvBL1. J. Biol. Chem..

[35] Khersonsky O., Tawfik D.S. (2010). Enzyme promiscuity: A mechanistic and evolutionary perspective. Annu. Rev. Biochem..

[36] Chapple C.E., Robisson B., Spinelli L., Guien C., Becker E., Brun C. (2015). Extreme multifunctional proteins identified from a human protein interaction network. Nat. Commun..

[37] Martínez-Núñez M.A., Rodríguez-Escamilla Z., Rodríguez-Vázquez K., Pérez-Rueda E. (2017). Tracing the repertoire of promiscuous enzymes along the metabolic pathways in Archaeal organisms. Life.

