# Supplementary material for: In Sulfolobus solfataricus, the Poly(ADP-Ribose) Polymerase-Like Thermoprotein Is a Multifunctional Enzyme
Source: Microorganisms. 2020 Oct 3;8(10):1523. doi: 10.3390/microorganisms8101523 (PMC7599888; doi:10.3390/microorganisms8101523)
Supplement: Supplementary file 1 [file microorganisms-08-01523-s001.pdf]

Supplementary Material

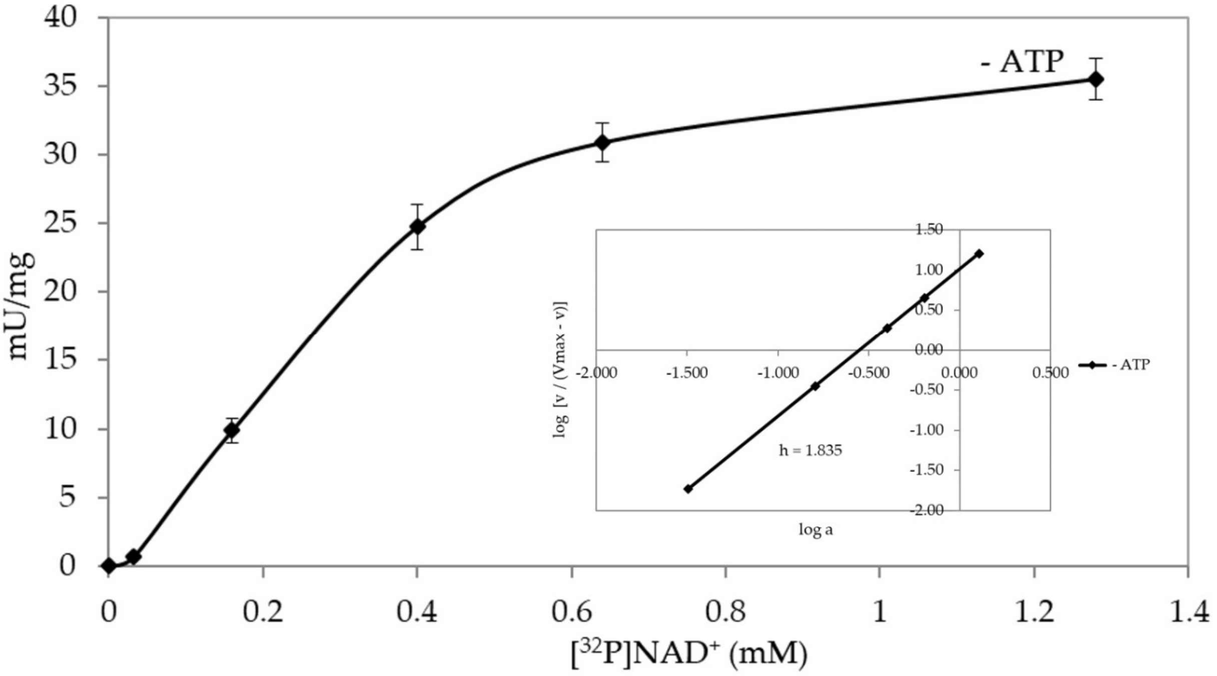

Figure S1. PARPSso Activity. The sigmoid curve was determined by measuring purified PARPSso activity at increasing concentrations of [^{32}P]NAD$^+$ (10,000 cpm/nmol). The reported values are means of 4 different assays with two enzyme preparations. The inlet shows the linearization of the curve by Hill equation:

$$\log [v/(V\text{-}1)] = h \log a - h \log K_{0.5}$$

Where V= Vmax; a= substrate concentration; $K_{0.5}$ = substrate concentration at half of Vmax; h= n_H= Hill coefficient measured by the slope of the plot. n_H=1.8.

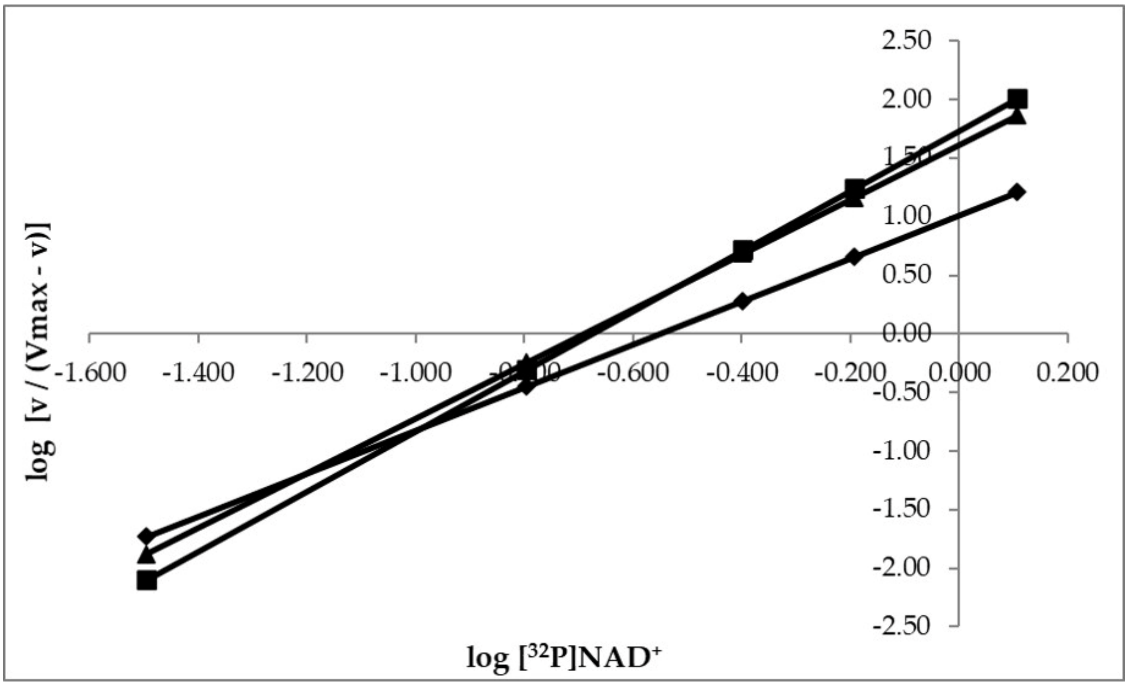

Figure S2. Linearized plots of ADPribosylating activity in presence of different ATP concentrations (μM: 0, ◆; 5, ■; 10, ▲). At 0.4mM [^{32}P]NAD$^+$ Ki was -7.9·10^{-3} (at 5 μM ATP), and -1.710^{-2} (at 10 μM ATP).

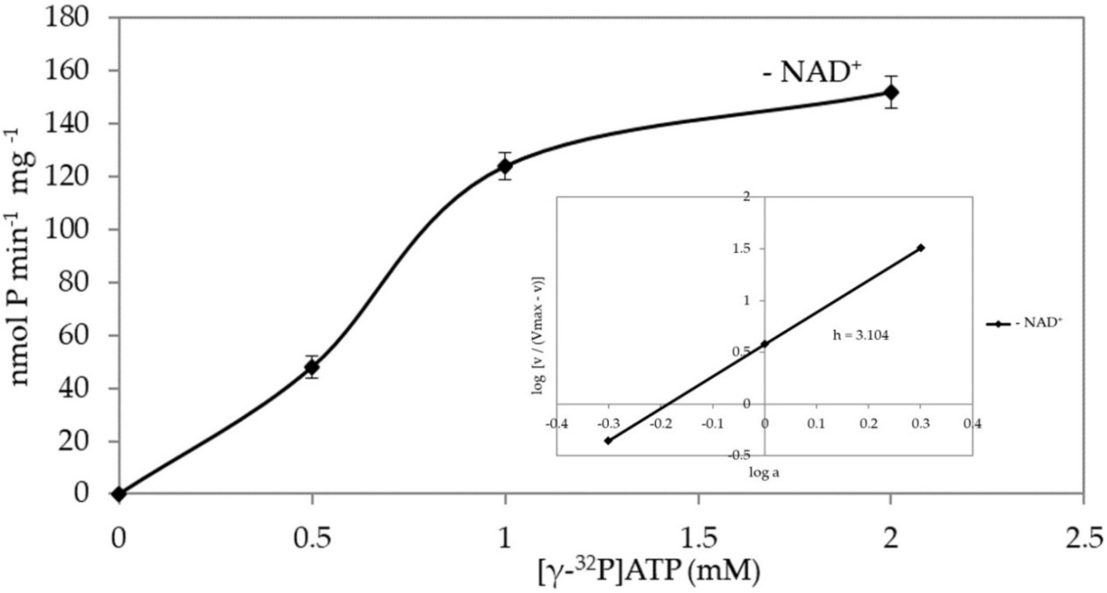

Figure S3.ATPase Activity of PARPSso. The sigmoid curve was determined by measuring purified PARPSso activity at increasing concentrations of[γ-^{32}P]ATP (10,000 cpm/nmole). The reported values are means of 4 different assays with two enzyme preparations.The inlet shows the linearization of the curve by Hill equation as described in the legend of Figure S1. n_H=3.1.

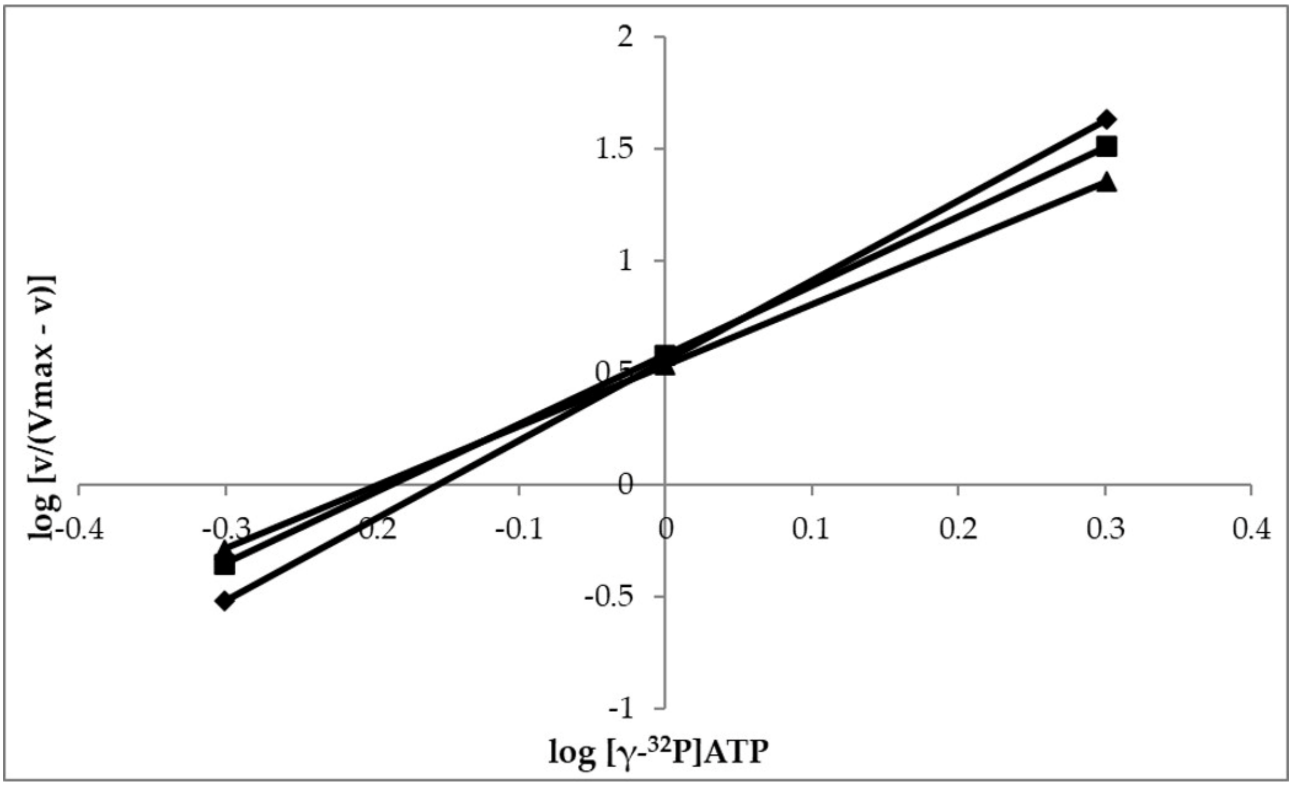

Figure S4. Linearized plots of ATPase activity in the presence ofdifferent NAD^+ concentrations (μM: 0, ■; 10, ◆; 100, ▲). At 0.5mM [^{32}P]ATP Ki was $7.3{\cdot}10^{-3}$ (at 10 μM NAD^+), and 1.910^{-1} (at 100 μM NAD^+).

Ki was calculated by the following equation:

$$1/v= [Km/(Vmax\ Ki\ [S])]\ [I] + 1/Vmax\ [1+ (Km/[S])]$$
